# 3D visualization of movements can amplify motor cortex activation during subsequent motor imagery

**DOI:** 10.3389/fnhum.2015.00463

**Published:** 2015-08-20

**Authors:** Teresa Sollfrank, Daniel Hart, Rachel Goodsell, Jonathan Foster, Tele Tan

**Affiliations:** ^1^Department of Psychology I, Institute of Psychology, University of WürzburgWürzburg, Germany; ^2^Department of Mechanical Engineering, Curtin UniversityPerth, WA, Australia; ^3^Department of Psychology and Speech Pathology, Curtin UniversityPerth, WA, Australia; ^4^Neurosciences Unit, Health Department of Western AustraliaPerth, WA, Australia

**Keywords:** brain-computer interfaces, EEG, motor cortex activation, 3-dimensional visualization

## Abstract

A repetitive movement practice by motor imagery (MI) can influence motor cortical excitability in the electroencephalogram (EEG). This study investigated if a realistic visualization in 3D of upper and lower limb movements can amplify motor related potentials during subsequent MI. We hypothesized that a richer sensory visualization might be more effective during instrumental conditioning, resulting in a more pronounced event related desynchronization (ERD) of the upper alpha band (10–12 Hz) over the sensorimotor cortices thereby potentially improving MI based brain-computer interface (BCI) protocols for motor rehabilitation. The results show a strong increase of the characteristic patterns of ERD of the upper alpha band components for left and right limb MI present over the sensorimotor areas in both visualization conditions. Overall, significant differences were observed as a function of visualization modality (VM; 2D vs. 3D). The largest upper alpha band power decrease was obtained during MI after a 3-dimensional visualization. In total in 12 out of 20 tasks the end-user of the 3D visualization group showed an enhanced upper alpha ERD relative to 2D VM group, with statistical significance in nine tasks.With a realistic visualization of the limb movements, we tried to increase motor cortex activation during subsequent MI. The feedback and the feedback environment should be inherently motivating and relevant for the learner and should have an appeal of novelty, real-world relevance or aesthetic value (Ryan and Deci, 2000; Merrill, 2007). Realistic visual feedback, consistent with the participant’s MI, might be helpful for accomplishing successful MI and the use of such feedback may assist in making BCI a more natural interface for MI based BCI rehabilitation.

## Introduction

Over the past several years, advances in the analysis of electroencephalogram (EEG) signals and improved computing capabilities have enabled people with severe motor disabilities to use their own brain activity for communication and control of objects in their environment, thereby bypassing their impaired neuromuscular system (Kübler et al., 2001; Wolpaw et al., 2002; Allison et al., 2007; Perdikis et al., 2014). A new potential brain-computer interface (BCI) therapeutic approach that is generating substantial interest concerns the use of EEG-based BCI protocols to improve volitional motor control that has been impaired by trauma or disease by influencing processes underlying brain plasticity and thereby inducing recovery of motor control (Pichiorri et al., 2011). Repetitive movement practices by motor imagery (MI) can influence activity-dependent central nervous system (CNS) plasticity that underpins normal function. For example, it has recently been suggested that MI based BCI training can restore motor control in persons with hemiplegia due to stroke (Daly and Wolpaw, 2008; Broetz et al., 2010; Caria et al., 2011). Pichiorri et al. (2011) showed that training with MI led to a significant increase in motor cortical excitability in BCI naïve participants. The peak amplitude and volume of the motor evoked potentials recorded from a particular hand muscle were significantly higher only in those subjects who developed a MI strategy based on imagining their hand grasping in order to successfully control a computer cursor. Furthermore, functional analysis indicated that there was a change in the topology of active brain networks with practice of hand grasping MI. Rizzolatti et al. (2001) suggested that the capacity to associate the visual representation of an observed action with the motor representation of that action can lead to imitative learning. By inducing a better engagement of motor areas with respect to MI, it has therefore been suggested that BCI protocols are able to influence and guide neuroplasticity to promote recovery in affected brain regions to restore motor function after brain injury (Mulder, 2007; Cincotti et al., 2012).

Sensorimotor rhythms (SMRs) refer to localized sinusoidal frequencies in the upper alpha band (10–12 Hz; Pfurtscheller and Neuper, 2001), which can be recorded over primary somatosensory and motor cortical areas. SMR decreases or desynchronizes (event related desynchronization, ERD) by movement, observing the movement of others and by imagined self-movement (MI) in the contralateral sensorimotor areas (Schnitzler et al., 1997; Lotze et al., 1999; Neuper et al., 2005; Halder et al., 2011). MI is defined as the mental simulation of a kinesthetic movement without overt movements by muscular activity (Decety and Ingvar, 1990; Neuper et al., 2005, 2009). Signal processing algorithms, individual user’s characteristics, such as psychosocial and physiological parameters (e.g., fine motor skills) or brain structures, can predict performances for SMR-based BCIs (Blankertz et al., 2010; Halder et al., 2011; Hammer et al., 2012; Randolph, 2012). Besides these factors, feedback is a necessary feature for initial learning to modulate the sensorimotor rhythm (Wolpaw et al., 1991, 2002; McFarland et al., 1998). The end-user have to be properly trained to be able to successfully control their EEG signals, especially for the use of a BCI based on the recognition of mental imagery tasks (e.g., MI; Neuper and Pfurtscheller, 2001).

To learn modulating SMR power, usually unimodal visual feedback is provided: the end-user receives feedback by an extending bar or a moving cursor in one or two dimensions according to the classification results (Neuper and Pfurtscheller, 2010; Schreuder et al., 2010). This feedback can often be wrong, due to a poor performance in the calibration task, since first time end-users cannot be expected to perform the required mental tasks perfectly from the start (Lotte et al., 2013). The feedback and the feedback environment should be inherently motivating and relevant for the learner and should have an appeal of novelty, challenge, real-world relevance or aesthetic value (Ryan and Deci, 2000; Merrill, 2007). This supports the use of more engaging feedback environments, employing rather realistic and engaging feedback scenarios, which are closely related to the specific target application. A rich visual representation of the signal e.g., in the form of a 3-dimensional video game or Virtual reality (VR) environment may enhance the end user’s control of a SMR based-BCI (Pineda et al., 2003). Subjects learned to control levels of SMR activity and were able to control a SMR-based BCI (Pfurtscheller et al., 2006a; Friedmann et al., 2007) during motivationally engaging and a realistic, interactive task. On the basis of these and related findings, some researchers have proposed that realistic feedback is a powerful medium to improve BCI-presentation by creating immersive and motivating environments (Friedmann et al., 2007; Leeb et al., 2007; Ron-Angevin and Díaz-Estrella, 2009). This may also be expected to help the end-user getting used to richer and more complex environments, thus lowering the mismatch between the feedback provided during training and during real-world use (Lotte et al., 2013). For example one could expect that observing a realistic moving hand should have greater effect on the SMRs than watching an abstract feedback (Pfurtscheller et al., 2007).

For the successful restoration of CNS function via the use of SMR-based BCI as a rehabilitation tool, interventions that optimally induce activity-dependent CNS plasticity must be developed. In the current context, the most effective kind of feedback visualization must be properly identified in order to enhance standard care approaches for the rehabilitation of motor function, which typically focus on interventions involving the upper and lower limbs (Mulder, 2007; Daly and Wolpaw, 2008). On this basis, the current study investigated if a 3-dimensional visualization of five different upper and lower limb movements could amplify motor cortex activation during subsequent MI and thereby give prospective support for the use of a SMR based BCI. The purpose of this study was therefore to identify possible advantages associated with the use of an enriched 3-dimensional movement visualization as opposed to the use of 2-dimensional modality. We hypothesize that this type of “realistic” and more sensorial rich visualization might be more effective during instrumental conditioning, in which the EEG signal classifier is fixed and unknown to the end-user, and this user has to find out how to control a cursor by modulating the brain activity in a specific way, resulting in more pronounced ERD of the SMR rhythm (10–12 Hz) over the sensorimotor cortices. Therefore we conducted a controlled study design with healthy volunteers to identify the reactivity of SMRs during MI after showing realistic 2D and 3D limb movement video presentation.

## Materials and Methods

### Participants

In total, 39 healthy SMR-BCI novices took part in the study which was approved by the Human Research Ethics Committee of the Office of Research and Development at Curtin University. Each participant was informed about the purpose of the study and signed informed consent prior to participation. Four of the participants were excluded from analysis due to noise in the data: three of them were moving too much during the experiment and for one it was not possible to attain impedances lower than 20 kΩ. Of the 35 participants whose data were included in the final analysis, 18 were women and the mean age of the sample was 26.56 years (SD 5.33, range 18–54). Two participants were left-handed. All participants had normal or corrected-to-normal vision.

### Experimental Set-Up

**Figure 1:**
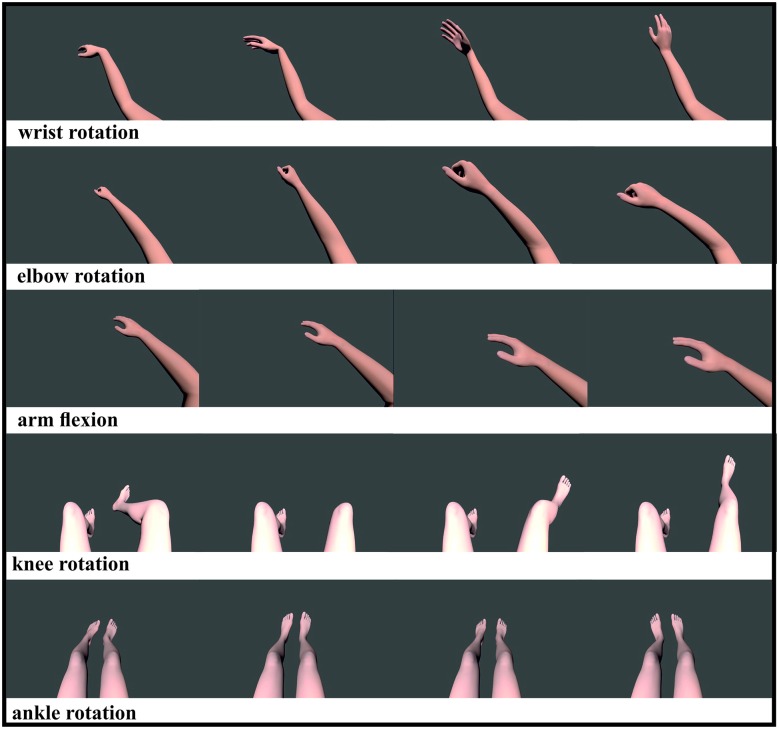
**Visualization of five different limb movements: wrist movement, elbow rotation, arm flexion, knee and ankle rotation**. All movements were shown for the left and right limb, except the ankle rotation which showed both feet rotating simultaneously. All videos were displayed randomized in 2D and 3D.

Participants were seated in a comfortable chair directly in front of a True3Di 24″ SDM-240M Stereoscopic 3D Monitor wearing stereoscopic glasses. Each participant’s chin lay on a pre-assembled chin holder. Participants were instructed to sit in a relaxed posture with their eyes open and avoiding any eye and body movements. Using a within subjects design, all participants were instructed to watch attentively 18 randomized videos of different limb movements for the left and right body part that were presented on a stereoscopic screen. Videos were displayed in 2D and 3D (Figure 1), portraying the following movements of computer-generated models: rotation of the wrist, elbow, knees and ankle anteriorly and an arm flexion towards the spectator. The videos displayed the movements from the perspective of the participant to encourage the feeling that each participant was moving their own limbs. At the end of each video a 6 s recording phase started, with a blank screen being presented during this phase. During this recording period, participants were requested to replicate subsequently the just observed movement by MI. The task was to perform a kinesthetic rather than visual MI (Neuper et al., 2005). Instructions were important during this experiment, as the participants only received offline feedback. Participants were instructed to feel the just observed motion in their muscles and they should vividly remember a situation in which they performed a given movement before imagining it during the subsequent BCI use. This should activate their prior experience with the task they will imagine, which is expected to make the learning easier (Merrill, 2007). Data collection lasted 45 min, with participants performing three runs of 10 min each, with 5 min breaks between each run.

### Data Acquisition

The EEG was recorded from 40 channels located over the sensorimotor cortex. The locations of the Ag/AgCl electrodes were based on the modified 10–20 system of the American Electroencephalographic Society (Sharbrough, 1991). Each channel was referenced to the left and grounded at the right mastoid. Impedances were kept below 5 kΩ via application of conductive gel. Data were collected via Neuroscan EEG equipment and signals were amplified using NuAmps amplifier. Data were sampled at 1000 Hz and bandpass filtered between 0.1–70 Hz with an additional notch filter applied to remove 50 Hz noise. A program algorithm was written to determine the presence of eye-blink artifacts; if identified, data from these periods were deleted. Data processing and storage were performed on a conventional laptop with an additional external monitor.

### ERD/ERS Analyses

EEG signals were visually inspected and trials contaminated with muscle or eye movement activity were discarded. ERD/ERS (Energy Recovery System) calculation was undertaken by bandpass filtering of each trial, squaring of samples and subsequent averaging over trials and over sample points (Graimann et al., 2002). The ERD/ERS were expressed as proportional power decrease (ERD) or power increase (ERS) of the imagery period in the upper alpha frequency band (10–12 Hz) and were calculated relative to the baseline, in relation to a 1 s reference interval before the imagery period started. We generated topographical maps averaged for all participants for each task and visualization modality (VM). The resulting maps represent plots of significant ERD within the given frequency range of 10–12 Hz. Based on the results of the topographical maps, we computed the mean ERD/ERS in the alpha frequency band (10–12 Hz) with the traditional ERD/ERS method proposed by Pfurtscheller and Lopes da Silva (1999). For statistical analyses, we used the ERD/ERS values obtained from the right (C4) vs. left sensorimotor cortex (C3) temporally aggregated over the imagery period (1–6 s). In order to analyze the potential influence of the VM on the ERD/ERS patterns during task performance we performed a repeated measures ANOVA using the VM, task, electrode position (EP) and task side as within-subjects variables. The probability of a Type I error was maintained at 0.05.

## Results

**Figure 2:**
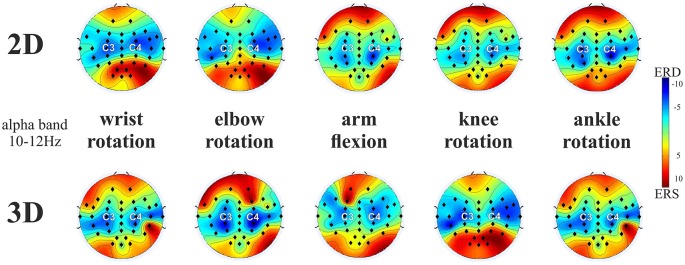
**ERD/ERS patterns averaged over all end-users for the five motor imagery (MI) tasks (averaged across left and right limb movements) for 2D and 3D visualization modality (VM) in the upper alpha frequency band (10–12 Hz)**. Note: ERD is indicated in blue and ERS is indicated in red. The black dots represent the electrode positions (EPs).

Figure 2 compares the topographical maps of the mean ERD values for the two VM groups, separately for the respective tasks (rotation of the wrist, elbow, knees and ankle in front and arm flexion towards the spectator) and pooled for both left and right MI in the upper alpha frequency band (10–12 Hz). In general, the results show a strong increase of the characteristic patterns of sensorimotor ERD of the upper alpha band components for left and right limb MI present over the sensorimotor areas in both visualization conditions. On basis of these findings EPs C3 and C4 were selected for further analyses, which is in accordance to other MI studies (Ron-Angevin and Díaz-Estrella, 2009; Neuper et al., 2009; Ono et al., 2013). A repeated measures ANOVA was performed on the ERD/ERS data using the VM (2 levels: 2D vs. 3D), task (5 levels: wrist movement, elbow rotation, arm flexion, knee and ankle rotation), EP (2 levels: C3 vs. C4) and task side (2 levels: left vs. right) as within-subjects variables, in order to analyze the potential influence of the VM on the ERD patterns during MI. In addition, we performed two 5 × 2 × 2 ANOVAs using the variables task, EP and task side as within-subjects variables for the two VM groups separately. Table 1 provides an overview of the significant ANOVA effects. Overall, significant differences were observed as a function of VM. This main effect is primarily due to the larger ERD during MI after 3D feedback. The significant main effect of Task indicates that ERD varied upon the different tasks. The averaged data for all upper limb (wrist rotation, elbow rotation, arm flexion) and lower limb MI tasks (knee rotation, ankle rotation) separated for the 2D and 3D condition were checked for normal distribution. Afterwards a *post hoc* paired sample *t*-test revealed significant smaller ERD values for lower limb MI tasks compared to upper limb MI tasks for the 2D (*t*_(368)_ = 3.74, *p* = 0.041) and for the 3D (*t*_(368)_ = 4.21, *p* = 0.0433) VM. A significant interaction between EP and task was found, which established the contralateral dominance of ERD. This analysis revealed significant interactions involving the factors VM, task, EP and task side (Table 1). *Post hoc* paired *t*-test comparison indicated that the largest upper alpha band power decrease during MI was obtained subsequent to the 3-dimensional visualization averaged or all tasks and both EPs (*t*_(1007)_ = 3.126, *p* = 0.002).

**Table 1:** **Summary of significant *F*-values^a^ for ERD/ERS analyses for the whole sample and separated for each visualization modality (VM)**.

	ANOVA effects		
	Whole sample (*n* = 35) VM (2) × Task (5) × EP (2) × Task Side (2)	2D VM (*n* = 35) Task (5) × EP (2) × Task Side (2)	3D VM (*n* = 35) Task (5) × EP (2) × Task Side (2)
VM	*F*_(1.73)_ = 20.48**
VM × Task	*F*_(1.73)_ = 9.12**
VM × EP	*F*_(1.73)_ = 8.54**
VM × Task × EP	*F*_(1.73)_ = 4.57**
VM × Task × Task side	*F*_(1.73)_ = 4.32*
Task	*F*_(1.73)_ = 6.90**	*F*_(1.73)_ = 2.69*	*F*_(1.73)_ = 12.51**
Task × EP	*F*_(1.73)_ = 2.95**		*F*_(1.73)_ = 5.81**
Task × Task Side	*F*_(1.73)_ = 4.72**		*F*_(1.73)_ = 6.89**
EP × Task Side	*F*_(1.73)_ = 4.08*		*F*_(1.73)_ = 4.78*
Task × EP × Task Side	*F*_(1.73)_ = 8.21**		*F*_(1.73)_ = 6.57**

^a^p-values 5% (*) and 1% (**). All repeated measures tests are Huynh-Feldt corrected. EP, Electrode position.

**Figure 3:**
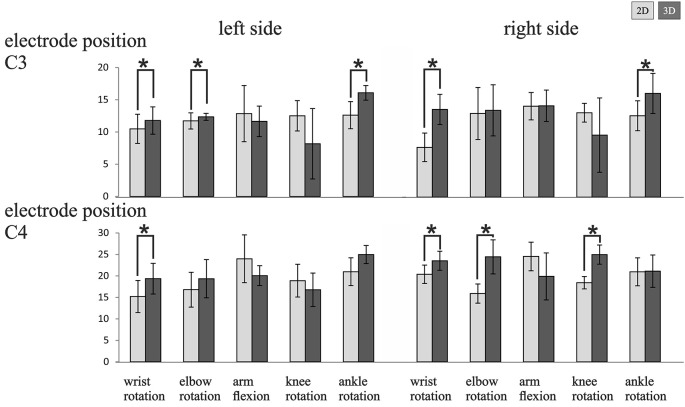
**Mean ERD/ERS values (i.e., mean and standard deviation) obtained for the left (left panel) and right (right panel) limb MI side of the 10–12 Hz upper alpha frequency band for all subjects with the two visualization conditions (2D, light grey bar; 3D, dark grey bar) on EP C3 and C4**. Significant differences between the visualization modalities are indicated (*p < 0.01).

Figure 3 presents a detailed overview of the mean ERD/ERS values with standard deviation and with *t*-test *post hoc* comparisons using a conservative significance level of 0.01, since we did not correct for multiple comparisons for the two visualization modalities (2D and 3D), separately for the different task, task side (left and right MI) and EP (C3 and C4). A difference between the visualization modalities can be seen in almost all tasks, depending on the EP and side of movement. In total in 12 out of 20 tasks the end-user of the 3D visualization group showed an enhanced upper alpha ERD relative to 2D VM group, with statistical significance (although not corrected for multiple comparisons) in nine tasks. The pattern of results suggests a generally higher ERD over the right (as compared to the left) sensorimotor region.

## Discussion

The present study was performed to investigate whether a 3-dimensional visualization of upper and lower limb movements can amplify motor cortex activation during a subsequent MI phase. Little is currently known about the impact of such a “realistic” VM. The SMR rhythm in humans can characteristically be found over the sensorimotor area with peaks around 10–12 Hz (Kuhlman, 1978; Hari et al., 1998; Pfurtscheller and Neuper, 2001). This frequency shows typical reactivity in association with MI (Pfurtscheller and Neuper, 1997; McFarland et al., 2000; Wolpaw et al., 2002; Blankertz et al., 2010). In the present study, we found a discernable decrease of the upper alpha rhythm (10–12 Hz) during imagery of limb movements over sensorimotor areas that significantly increased in the 3D visualization condition. The results showed in both VM conditions a more pronounced ERD for MI of the upper limbs compared to the lower limbs. We think this could be explained with the fewer difficulties that the SMR naïve participants have in imagining hand and arm movements. In daily life, we pay more attention to our movements of the upper limbs than conscious movements with the foot or knees and could explain the effect on motor cortex activation during MI.

The visualization of the different limb movements in a first person perspective was supposed to facilitate the task of performing MI. One potential limitation of the realistic video presentation was due to the fact that we used computerized limb models. We tried to create them as realistic as possible with skin color, texture and anatomical correct movement sequences. Especially for rehabilitation a computer animated version can give the advantage to adapt the limb to each individual user. Although a lot of effort was contributed in video programing still a visible difference exists compared to a video of a real limb movement. We refrained from using videos of taped limb motion, as this would not be an option for impaired patients. The main difficulty some people have especially those with limited motor function is to get a kinesthetic feeling (Neuper et al., 2005) of the movements and we wanted to support their MI by showing the specific motion beforehand. Previous work has suggested an important role for the perception of the body within a 3-dimensional environment (Slater et al., 1995). The body should be used naturally and should be anchored into the feedback for a successful ERD reproducibility. A possible explanation for this effect is the activation of the SMR which is in correspondence to the human mirror neuron system. This system matches action observation and execution and is capable of performing a simulation of just observed actions (Pineda, 2005; Neuper et al., 2009) and some researchers proposed a functional link between the observation of an action, the internal simulation, MI and the execution of the motor action (Grèzes and Decety, 2001; Neuper et al., 2005). The execution, imagination or observation of motor actions produces asynchronous firing in the mirror neurons and causes a suppression or desynchronization of the SMR-rhythm (Lopes da Silva, 2006). To exclude an overlaying effect of “motion observation” on the ERD in the alpha band (Muthukumaraswamy et al., 2004; Hammon et al., 2006; Perry and Bentin, 2009) we integrated a short pause between the videos and the MI phase, where the screen turned blank. How long the ERD of such a motion observation can last is not yet known. To be sure that the effects on the upper alpha band are only due to actual MI, we expanded the MI phase to 6 s. The current findings indicate that a 3-dimensional realistic presentation of movements to support a subsequent MI phase seems to be a suitable strategy to achieve locally restricted activation patterns for SMR-BCI use.

In a study by Friedmann et al. (2007), participants tried to control a SMR-based BCI in a CAVE system and showed that navigation was possible. Participants reported afterwards that they were more motivated in this kind of task compared to the training on a conventional visual monitor. They also reported that the interaction seemed more natural to them than traditional BCI. Virtual reality and 3D non-VR visualization are powerful tools with significant possibilities to improve BCI-feedback presentation (Pineda et al., 2003; Pfurtscheller et al., 2006b; Ron-Angevin and Díaz-Estrella, 2009). With this technology immersive and motivating environments can be created, which can positively influence a successful training (Leeb et al., 2007). A study by Gruzelier et al. (2010) could show that a SMR neurofeedback training in VR could enhance the artistic performance of actors more successfully than a training with a 2D feedback rendition. The efficacy of this training was attributed to the psychological engagement through the ecologically relevant learning context of the immersive VR technology.

We could show that the 3-dimensional visualization enhanced ERD in the upper alpha band in some but not in all MI tasks. Eleven tasks showed no significant differences in the mean ERD values however a high variance in this data can be found. A study by Neuper et al. (2009) compared the effects of abstract and realistic feedback on SMR BCI performance and could not find any significant differences between the two groups. One explanation for that was that feedback stimuli seem to become closely associated with the action goal during MI and therefore both feedback types were able to enhance the desired electrophysiological signals for individuals to perform accurately. This could also be true for our experiment. Most of the present studies compared “abstract” vs. “realistic” feedback (Neuper et al., 2009), presented activation maps during BCI training (Hwang et al., 2009) or game like feedback in VR (Scherer et al., 2008; Ron-Angevin and Díaz-Estrella, 2009; Zhao et al., 2009). Our study compared for the first time the actual effects of 2D and 3D visualization on MI during the same limb motion tasks: the video of the movements of the limbs were the same in both visualization conditions. We were aware that differences in the motor cortex activation may only be slightly detectable due to the similarity of the video presentations. In future studies, the influence of these two visualization modalities have to be further investigated as it is possible that the effect can be increased in an online setting where the end-user imagined movements affect the animated limb in real time. Following the herein presented results we can conclude that VM plays an important role in a SMR-controlled BCI. Providing end-users with a realistic 3-dimensional presentation of limb movements seems to help to get a concrete feeling of kinesthetic MI and exerts significant effects on motor cortex activation.

## Conflict of Interest Statement

The authors declare that the research was conducted in the absence of any commercial or financial relationships that could be construed as a potential conflict of interest.
